# The Mad River Dental Society

**Published:** 1871-06

**Authors:** 


### THE MAD RIVER DENTAL SOCIETY

Met on Tuesday, May 9th, at Dayton. There was quite a large attendance, and a very interesting meeting was held. Several essays were read, and much interesting discussion was had upon them, and also several topics discussed upon which no papers were read.

The subject of professional education elicited a very general and interesting discussion; high ground was taken by all the members. An unmistakable desire was expressed that our colleges should take higher ground in the matter of training young men for the profession.

Many new modes and appliances were presented that were valuable, interesting and instructive. The Mad River Dental Society is one of the active societies of the State, and should have in its membership, every active member of the profession in the south-western part of the State. The next meeting is to be held at Yellow Springs, on the second Tuesday of September next, when it is expected there will be a large meeting; the place is delightful, and one that will induce many to bring their wives with them. It is proposed to continue the meeting two days, and intersperse social enjoyment with professional work, then let all come with this end in view. T.

